# A Case of Bilateral Infected Kidney Stones Presenting With Septic Shock and Acute Kidney Injury

**DOI:** 10.7759/cureus.22506

**Published:** 2022-02-22

**Authors:** William Lim, Maham Suhail, Keith Diaz

**Affiliations:** 1 Internal Medicine, Richmond University Medical Center, Staten Island, USA; 2 Pulmonary and Critical Care, Richmond University Medical Center, Staten Island, USA

**Keywords:** urinary tract infection, bilateral kidney stones, acute kidney injury, septic shock, urolithiasis

## Abstract

The incidence of unilateral ureteral calculi has been reported at 20% in the literature; however, cases of bilateral kidney stones are not commonly reported in the urology, nephrology, and emergency medicine literature. Here, we present the case of a 31-year-old female who presented to the emergency department with septic shock and acute kidney injury. Acute kidney injury was initially thought to be prerenal secondary to septic shock but later found to have complete bilateral ureteral obstruction as a cause of septic shock and acute kidney injury. Urinary tract infection and sepsis secondary to bilateral obstructing ureteral stones are some of the few true urological emergencies. Anuria is the common presenting feature of complete urinary tract obstruction, and physicians should have a high index of suspicion for this diagnosis in anuric patients as rare as it is.

## Introduction

The incidence of unilateral ureteral calculi has been reported at 20% in the literature [1]; however, cases of bilateral kidney stones are not commonly reported in the urology, nephrology, and emergency medicine literature. Urinary tract infection (UTI) and sepsis secondary to bilateral obstructing ureteral stones are some of the few true urological emergencies and have a mortality rate of 7%. In such patients, urgent intervention is essential in limiting complications and improving patients’ outcomes. Percutaneous nephrolithotomy is a preferred intervention procedure because it is shown to have a higher stone-free rate and lower complications.

## Case presentation

A 31-year-old female with a medical history of anoxic encephalopathy with ventilator dependence, chronic viral hepatitis C, and type 2 diabetes mellitus presented from an assisted care facility to the emergency department (ED) for evaluation of high-grade fever, hypoxia, and tachypnea. Her vital signs were notable for the following: temperature, 101.8°F; blood pressure, 87/63 mmHg; pulse rate, 120 per minute; and respiratory rate, 22 breaths per minute. Her initial laboratory workup was significant for the following: white blood cell count, 23.8 k/µL with neutrophilic predominance; blood urea nitrogen, 30 mg/dL; creatinine, 2.3 mg/dL; and lactic acid, 3.9 mmol/L. A Foley catheter was placed, and the initial urine output was 10 cc. Urinalysis (UA) was positive for bacteria and leukocyte esterase and negative for nitrate. Urine pH was >9 and urine red blood cell count was 12/HPF and 17/HPF. The fractional excretion of sodium was calculated to be 0.9%. Empiric broad-spectrum antibiotics and intravenous (IV) lactated Ringer’s were administered. The patient’s blood pressure was not responsive after a trial of fluid (3 L) and IV norepinephrine was started. The patient’s acute kidney injury was presumed to be prerenal secondary to septic shock, for which maintenance fluids were started at a rate of 150 mL/hour. However, the patient continued to have a persistent high-grade fever of 104°F after 48 hours of broad-spectrum antibiotics with no urine output.

With the patient’s presentation of anuria, high-grade fever, UA showing UTI, and alkaline urine, ureteral obstruction with struvite stones secondary to urease-producing organisms was considered as the source of infection. Renal and bladder ultrasound (US) obtained on day two showed grade 2 mild bilateral hydronephrosis with 16 mm left interpolar renal calculus and no right-sided renal calculus. The patient’s renal function continued to worsen despite aggressive hydration; her serum creatinine increased from 2.3 mg/dL on admission to 4.6 mg/dL on day two and 6.2 mg/dL on day three.

**Figure 1:**
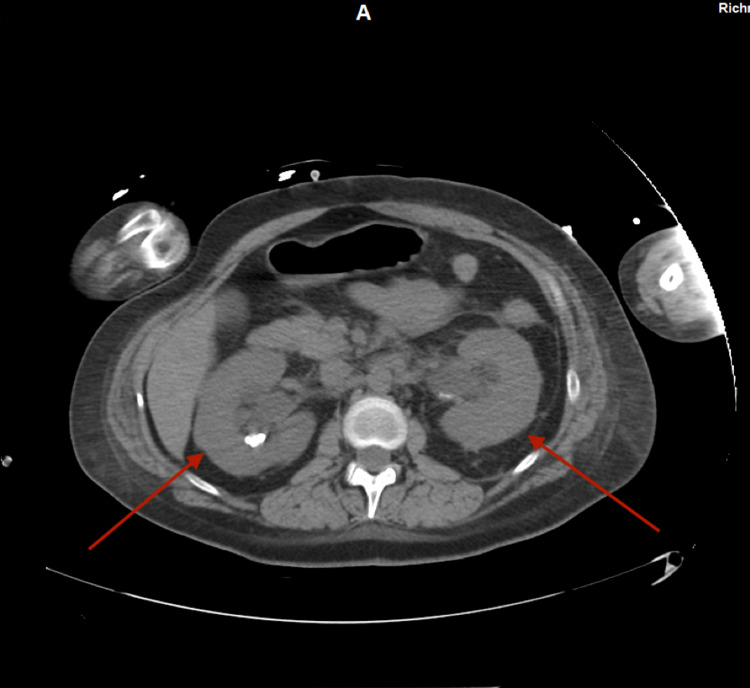
CT scan of the abdomen and pelvis showing bilateral hydronephrosis. CT: computed tomography

**Figure 2:**
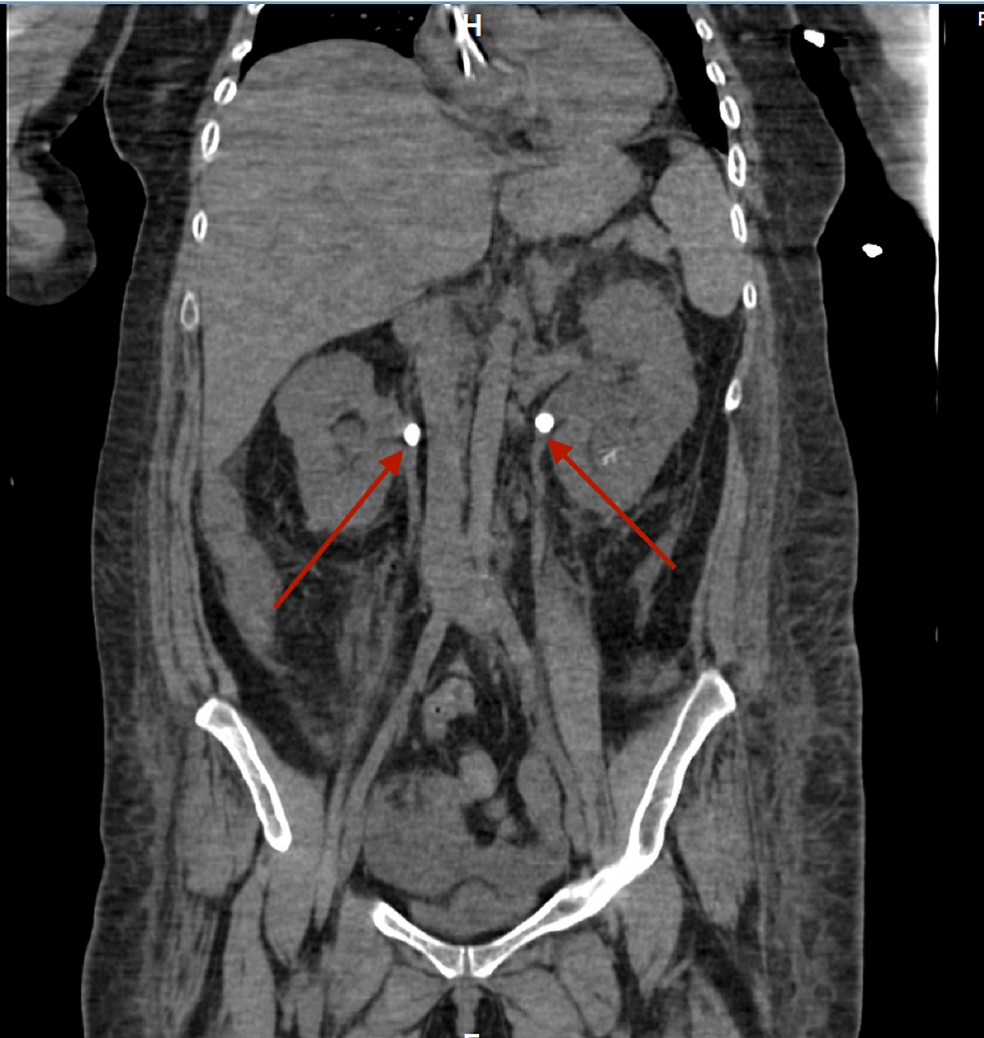
CT scan of the abdomen and pelvis showing bilateral obstructing stones at the ureteropelvic junction.

**Figure 3:**
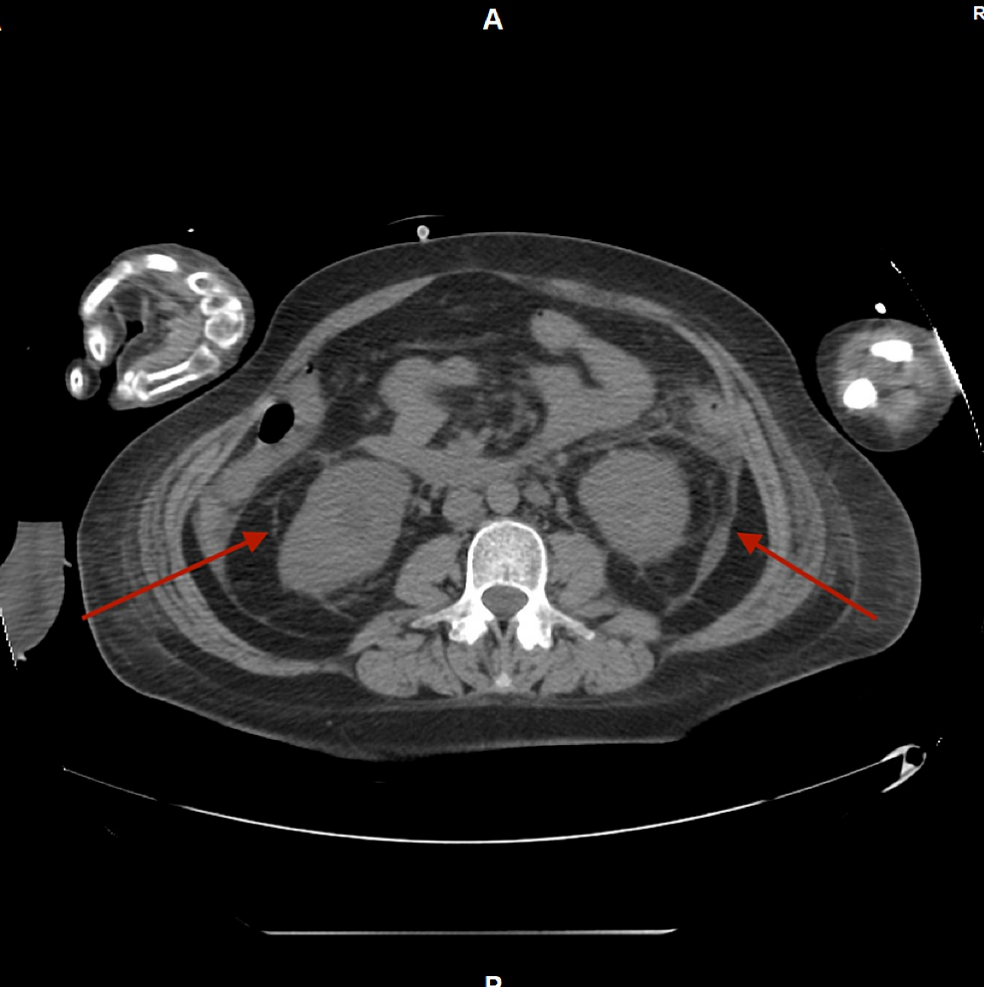
CT scan of the abdomen and pelvis showing bilateral perinephric fat stranding. CT: computed tomography

Given her persistent high-grade fever despite broad-spectrum antibiotics, worsening renal function, and anuria, complete bilateral ureteral obstruction was considered even though no obstruction was seen on the renal US. Computed tomography (CT) scan of the abdomen and pelvis without contrast was obtained which revealed bilateral hydronephrosis (Figure 1) secondary to ureteral obstruction with a 12 mm right-sided stone and a 10 mm left-sided stone at the ureteropelvic junction (Figure 2) with perinephric fat stranding (Figure 3).

Interventional radiology was consulted and a nephrostomy tube was placed for urgent drainage bilaterally. Over the next two days, the patient’s creatinine normalized and her urine output improved. On day four, the patient’s urine culture and blood culture came positive for *Proteus mirabilis*. Over the next few days, her condition improved, she was titrated off vasopressors, and her fever subsided. The patient was discharged to follow-up appointments with urology as an outpatient for definitive treatment of kidney stones.

## Discussion

Acute kidney injury due to postrenal obstruction is rare (<10%) because a single functioning kidney can compensate and maintain near-normal excretory function. Postrenal acute kidney injury develops almost exclusively in ureter obstruction with a single functioning kidney or in bilateral renal or lower urinary tract obstruction, which is most commonly caused by prostatic disease (hyperplasia or cancer) in men [2-4]. Rare causes such as retroperitoneal fibrosis can also cause progressive ureteral obstruction leading to acute kidney injury. Common clinical features of urinary tract obstruction include severe flank pain, changes in urine output, hypertension, and hematuria. Because our patient was nonverbal and could not communicate her symptoms, the diagnosis was more challenging.

The most common ureteral stones (urolithiasis) are calcium stones while other less common types include uric acid, cystine, and struvite stones [5]. Urease-producing organisms such as *Proteus mirabilis*, *Klebsiella pneumoniae*, *Corynebacterium* species, or *Ureaplasma urealyticum* break down urea into carbon dioxide and ammonia. Ammonia, in turn, combines with water to form ammonium leading to alkaline urine, which, in turn, decreases the solubility of phosphate leading to struvite stone formation. Diagnosis of struvite stones should be suspected if the patient has a history of recurrent or persistent UTIs, particularly those involving urease-producing organisms, persistently alkaline urine pH (>8), presence of magnesium ammonium phosphate crystals in the urine sediment, and staghorn calculus identified on abdominal imaging [6]. This patient’s clinical presentation of anuria, septic shock, and acute kidney injury together with alkaline urine pH and bacteriuria in UA indicated a UTI with urease-producing organisms.

With a sensitivity of 95% and specificity of 97%, a CT scan of the abdomen and pelvis without contrast is the imaging of choice for most patients with suspected nephrolithiasis [7]. CT scan can also detect secondary signs of ureteral obstruction such as ureteral dilatation and perinephric stranding (nonspecific signs pointing to an underlying inflammatory problem of the kidney and/or collecting system) [8,9]. Compared with CT scans, however, the sensitivity of US for detecting ureteric stones is significantly low at 45% [10,11]. This case highlights the significance of the CT scan versus the renal US in patients with suspicion of ureteral obstruction.

Urgent intervention is essential in patients with obstructed upper urinary tract system, which can reduce the entry of antibiotics into the collecting system by increasing renal pelvic pressure and leading to reduced glomerular filtration [12]. There are two options for urgent decompression, namely, either retrograde ureteral stent placement or percutaneous nephrostomy tube. Currently, there is no evidence to suggest that one modality is superior to the other [13].

There are various surgical options such as shock wave lithotripsy, percutaneous nephrolithotomy, laparoscopy, or open surgery based on the location and size of the stone and the patient’s situation. After surgical removal, prophylactic antibiotic therapy with either nitrofurantoin or trimethoprim-sulfamethoxazole is recommended for three to six months. Urease inhibitors such as acetohydroxamic acid can also be used in patients who have residual stone fragments following surgical stone removal of recurrent struvite stones because it can slow struvite stone growth by inhibiting urease production [14,15].

## Conclusions

Bilateral infected ureteral stones (urolithiasis) require emergent treatment to avoid catastrophic complications. Prompt recognition is essential to avoid irreversible damage to the kidneys. Anuria is the common presenting feature of complete urinary tract obstruction, and physicians should have a high index of suspicion for this diagnosis in anuric patients as rare as it is. This case highlights the role of anchoring bias and attributing acute kidney injury to be a prerenal etiology in patients with sepsis, which may have delayed diagnosis and treatment. It also points out the importance of interpreting UA, understanding the different radiological tests, and their sensitivities and specificity in the diagnosis of urolithiasis.
